# P79 - AsthmaVent – effect of mechanical ventilation on asthmacontrol in house dust mite allergic children with asthma

**DOI:** 10.1186/2045-7022-4-S1-P134

**Published:** 2014-02-28

**Authors:** Nina Viskum Hogaard

**Affiliations:** 1 Aarhus University Hospital, Aarhus, Denmark

## Background

House dust mite allergy is a frequent cause of asthma in children. Children with house dust mite allergy and asthma are especially sensitive to physical and chemical agents in the indoor air and as children spend a lot of time indoors, it seems reasonable to believe that improving the indoor environment will lead to an improvement in asthma disease. At present, there is no clear consensus on the effect of ventilation on asthmatic children with house dust mite allergy. Earlier studies have been criticized for being small, poorly carried out and inconclusive. Thus there is a need for a powerful and methodologically rigorous study to provide significant evidence as a basis for the future treatment of children with house dust mite allergy and asthma.

## Objective

This study started in the fall 2012 and aims at investigating whether mechanical ventilation is able to improve asthma symptoms during the winter season for children with house dust mite allergy and asthma.

## Materials and methods

The study is a randomized double-blind placebo-controlled intervention study, with 9 months of intervention and follow-up. 80 children are included from 3 Danish hospitals and are randomized into two groups. The intervention is mechanical ventilation in the child’s bedroom. We monitor indoor air quality and health outcomes every three months. Primary outcomes are minimal effective dose of inhalation steroids and reduction in levels of particulate matter and house dust mite allergen.

## Perspectives

Asthma patients and their families rely on good evidence-based advice on behavior and design of housing, so that the specific and non-specific factors in the indoor environment that trigger the disease are controlled as well as possible. The results of this project will be a significant contribution to the recommendations that can be given in relation to the effect of ventilation on indoor air quality in the asthma control of house dust mite allergic children.

